# Toxins and Antivenoms of *Latrodectus tredecimguttatus*: Molecular Mechanisms, Antibody Development, and Evidence from Other *Latrodectus* Species

**DOI:** 10.3390/ijms27209112

**Published:** 2026-10-14

**Authors:** Marziya Rakhim, Assima Abekova, Zhanserik Shynykul, Moldir Koilybayeva, Zhanat Batanova, Ernur Nurolda, Nurlan Akhmetsadykov

**Affiliations:** 1 The Faculty of Veterinary Medicine and Zooengineering, Kazakh National Agrarian Research University, Abay Avenue 8, Almaty 050010, Kazakhstan; marzi.95@mail.ru (M.R.); zhanat.batanova@gmail.com (Z.B.); 2 Research and Production Enterprise Antigen LTD, Karasai District, Abay Village, 4 Azerbayev Str., Almaty 040905, Kazakhstan; nurolda.ernur@gmail.com (E.N.); nurlan.akhmetsadykov@gmail.com (N.A.); 3 School of Pharmacy, Asfendiyarov Kazakh National Medical University, Tole-bi 94, Almaty 050012, Kazakhstan; moliya-90@mail.ru

**Keywords:** *Latrodectus tredecimguttatus*, alpha-latrotoxin, immunoglobulin, antivenom therapy, toxin neutralization, passive immunization

## Abstract

*Latrodectus tredecimguttatus* envenomation causes latrodectism, a neurotoxic syndrome driven primarily by α-latrotoxin (α-LTX)-mediated dysregulation of neurotransmitter release. This review examines the development and therapeutic application of specific immunoglobulins against *L. tredecimguttatus* toxins, integrating toxin diversity, α-LTX mechanisms, antibody production, preclinical neutralization, and clinical use. Eggs and spiderlings also contain bioactive proteins, including Latroeggtoxin-I and Latroeggtoxin-IV, although their relevance to human envenomation and antivenom development remains uncertain. α-LTX acts through receptor engagement, membrane pore formation, Ca^2+^ influx, and receptor-mediated signaling, providing potential targets for antibody neutralization. Conventional antivenoms rely mainly on animal-derived polyclonal immunoglobulins, whereas phage display and recombinant engineering enable fully human monoclonal antibodies with defined specificity and high affinity. Preclinical studies demonstrate neutralization across cellular, electrophysiological, ex vivo, and in vivo models, but methodological heterogeneity and variable cross-species activity complicate efficacy comparisons. Recombinant human antibodies offer improved molecular definition, reproducibility, and engineering potential, although clinical efficacy remains unestablished. Future development should prioritize standardized neutralization assays, clinically relevant post-exposure models, systematic cross-neutralization studies, and recombinant antibody combinations to achieve broader toxin coverage and safer, more standardized, reproducible, and clinically applicable immunotherapy for *L. tredecimguttatus* envenomation.

## 1. Introduction

**Figure 1:**
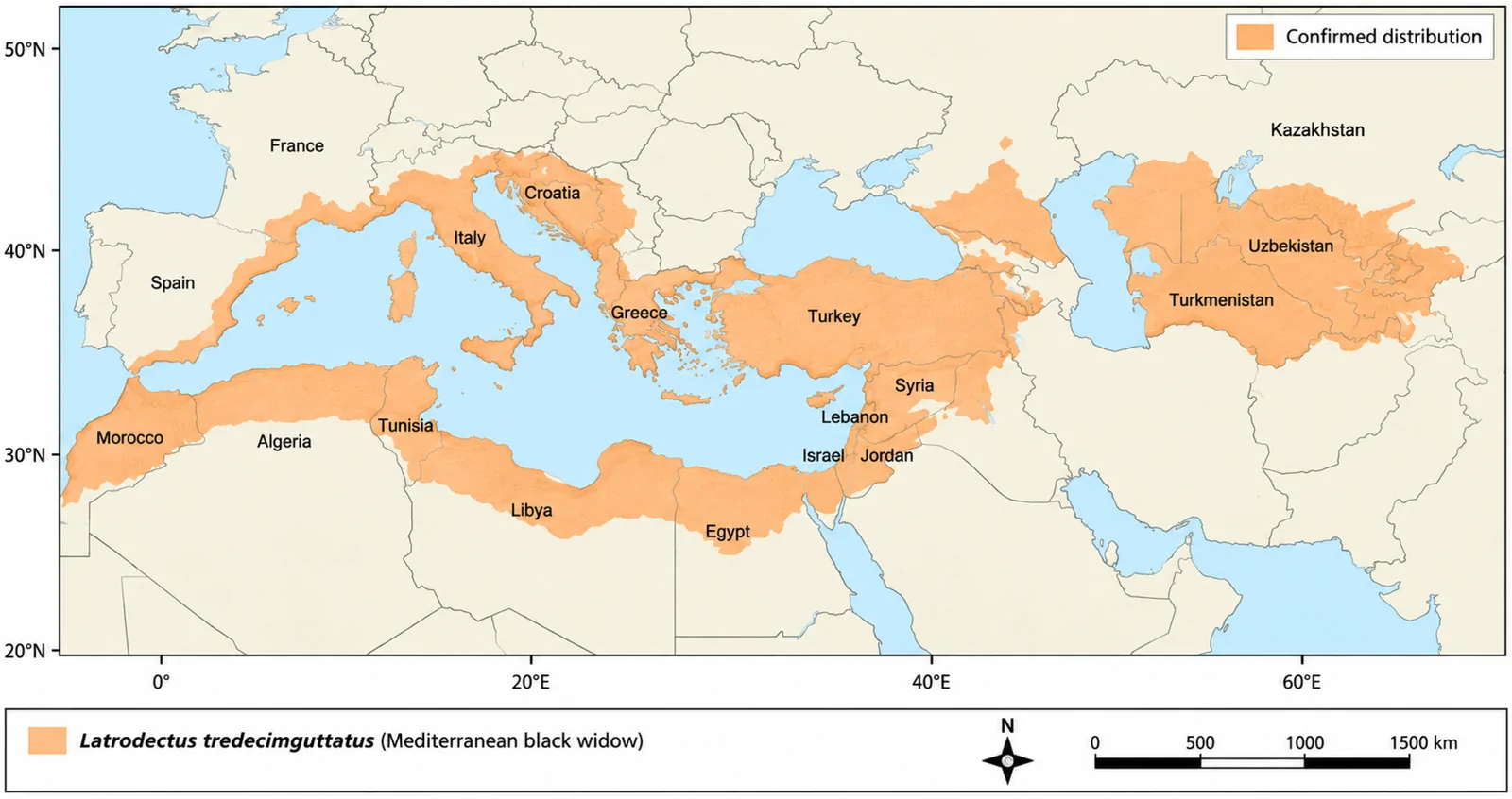
Geographic distribution of *L. tredecimguttatus* (original distribution map compiled from published sources [1,2,3,4]).

The genus *Latrodectus*, within the family Theridiidae (order Araneae, class Arachnida), comprises species distributed across Europe, Asia, the Americas, and Australia [1,2]. Among these, *Latrodectus tredecimguttatus*, known as the Mediterranean black widow, occurs in the Mediterranean region and parts of Eastern Europe and Asia, including Central Asia (Figure 1) [3,4]. *L. tredecimguttatus* inhabits dry, open environments, including agricultural areas, where human contact can result in medically significant envenomation [5].

Fatal outcomes following *Latrodectus* envenomation have been reported, but appear rare in the published clinical literature [6]. In a US poison-center study of symptomatic *Latrodectus* envenomation, 1.4% of cases had major clinical effects, but no deaths were recorded [7]. In a separate series of 163 black widow envenomations, generalized abdominal, back, and leg pain were common presenting complaints [8]. Direct clinical reports concerning *L. tredecimguttatus* also describe favorable outcomes. A clinical series concerning *L. tredecimguttatus* recorded 463 bites in Uzbekistan during 1981–1984 without a fatal outcome [9]. However, a case report described fatal toxic myocarditis following black widow spider envenomation [10]. These reports do not establish the mortality rate of untreated systemic envenomation. Incomplete reporting limits characterization of latrodectism epidemiology in the Americas [11]. Diagnostic uncertainty also requires caution when interpreting poison-center data on spider bites [12].

Latrodectism is the clinical syndrome caused by *Latrodectus* envenomation, with manifestations that vary among species and geographic regions [13]. Local pain and sweating are commonly reported following redback spider bites [14]. Muscular and abdominal pain are also prominent features of black widow spider envenomation [15]. Autonomic manifestations may include sweating and hypertension, while tachycardia has also been documented [8,16]. Severe presentations may involve generalized pain, muscle cramps or spasms, and abdominal muscle rigidity [17,18]. Although deaths are uncommon, severe pain and systemic manifestations may warrant urgent clinical assessment [7,8]. Recognition can be difficult in young children, in whom redback spider envenomation may present with nonspecific features such as irritability or persistent crying [19,20]. Hospital-based reports also document redback spider envenomation requiring inpatient care [21]. In a Central Australian retrospective series, Aboriginal patients received antivenom later than non-Aboriginal patients, and this delay was associated with more frequent sweating and fever [22].

Management of latrodectism generally relies on supportive care and symptomatic treatment, including analgesics and muscle relaxants. Animal-derived antivenoms provide a specific treatment option, but evidence of benefit depends on the implicated species, preparation, and study design. Findings from other *Latrodectus* species cannot independently establish clinical efficacy against *L. tredecimguttatus* [14,23,24,25]. Animal-derived antivenoms can cause immediate hypersensitivity reactions and delayed serum sickness [26]. Human antibody phage display libraries provide an alternative platform for antibody discovery [27]. The clinical benefits and adverse reactions associated with conventional antivenoms require careful assessment [28]. Recombinant antibody formats also require evaluation of immunogenicity; their design alone does not establish improved clinical safety [29].

The development of toxin-specific recombinant antibodies offers an alternative to antivenom production based on animal immunization, with the potential to produce preparations of defined antibody composition. Manufacturing analyses of recombinant snake antivenoms support investigation of this approach, but do not establish its clinical or economic advantages for *L. tredecimguttatus* [30]. Synthetic human antibody phage display libraries enable the selection and engineering of antigen-specific antibodies without animal immunization [31]. However, production costs depend on factors such as antibody potency, the required dose, and the manufacturing process; improved accessibility therefore cannot be assumed from recombinant design alone [32]. Toxin-specific antibodies may also support diagnostic assay development, for which analytical sensitivity, specificity, sample selection, and validation in envenomed patients are essential considerations [33]. For *L. tredecimguttatus*, α-LTX detection represents a potential application requiring dedicated validation, while improved clinical safety and therapeutic efficacy of recombinant preparations remain to be demonstrated.

This review critically examines toxin biology, molecular mechanisms, antibody development, preclinical neutralization, and clinical evidence relevant to *L. tredecimguttatus*. It distinguishes direct evidence for this species from experimentally demonstrated cross-neutralization and potentially informative findings involving other *Latrodectus* species. Conventional antivenoms and recombinant antibody candidates are evaluated according to their experimental support, limitations, and stage of development, with particular attention to the evidence required for clinical translation.

## 2. Toxins of *L. tredecimguttatus*

### 2.1. Known Venom Toxins

**Figure 2:**
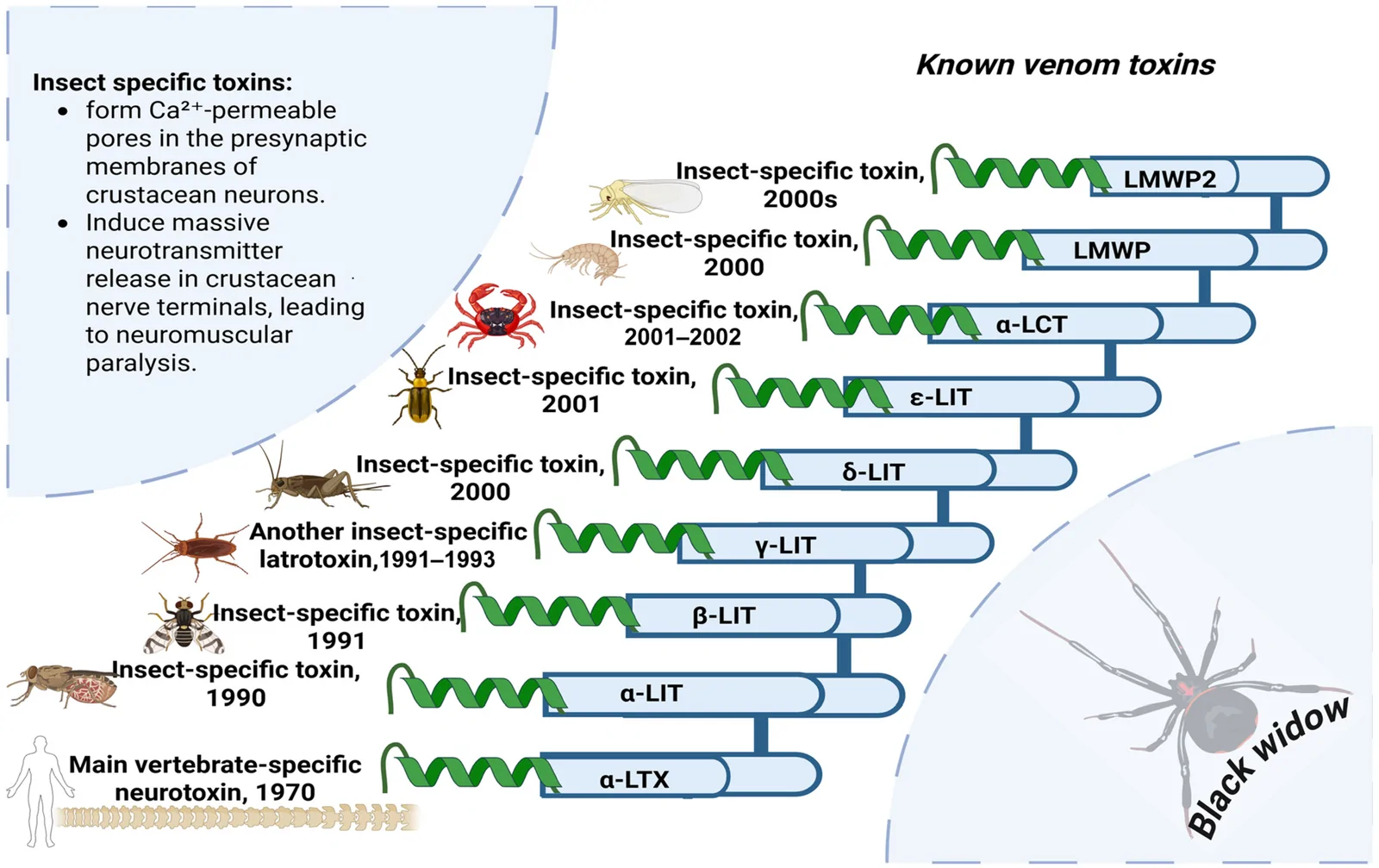
Diversity of known venom toxins in *L. tredecimguttatus*. The figure illustrates the chronological discovery and functional specialization of venom toxins isolated from the Mediterranean black widow spider (*L. tredecimguttatus*). Major toxins are classified based on their target specificity: vertebrate-specific α-latrotoxin (α-LTX); crustacean-specific α-latrocrustatoxin (α-LCT); insect-specific LTXs including α-latroinsectotoxin (α-LIT), β-latroinsectotoxin (β-LIT), γ-latroinsectotoxin (γ-LIT), δ-latroinsectotoxin (δ-LIT), and ε-latroinsectotoxin (ε-LIT); as well as two groups of low-molecular-weight peptides, LMWP (Low-Molecular-Weight Peptides) and LMWP2 (Low-Molecular-Weight Peptides 2). Insect-specific LTXs form Ca^2+^-permeable pores in presynaptic membranes of insect and crustacean neurons, leading to massive neurotransmitter release and neuromuscular paralysis (original schematic synthesized from published sources [35,36,37,38,43,44]).

To date, at least seven distinct LTXs have been described in the venom of *L. tredecimguttatus* [34,35] (Figure 2, Table 1). Most of these target invertebrates, particularly insects, and are classified as latroinsectotoxins (LITs), comprising α-, β-, γ-, δ-, and ε-LIT [34]. These proteins are large, with purified α-LIT having a reported molecular mass of approximately 120 kDa [36]. Experimental studies of α-LIT have demonstrated binding to insect nerve terminals and stimulation of neurotransmitter release [36], as well as formation of cation-selective channels in artificial lipid bilayers [37]. These findings should not be generalized to all LITs without toxin-specific evidence. Black widow spider venom has also shown toxicity to *Caenorhabditis elegans*, with susceptibility dependent on a latrophilin homologue; however, this finding does not establish that ε-LIT alone accounts for the observed toxicity [38]. Sequences encoding α-LIT and δ-LIT have been characterized through cloning and sequence analysis, providing insight into their molecular organization and evolution [39,40]. Recombinant expression in insect cells has enabled functional characterization of selected black widow spider neurotoxins [41]. In addition to these large toxins, a low-molecular-weight protein that co-purifies with α-LTX has been characterized and shown to be structurally related to crustacean hyperglycemic hormones [42]. Further biochemical characterization identified two low-molecular-weight venom proteins: LMWP and LMWP2 [43].

Among the characterized latrotoxins, α-LTX is the vertebrate-specific toxin [35]. Its mechanism entails attaching to presynaptic neuronal receptors, such as neurexin and latrophilin, and creating calcium-permeable pores in the neuronal membrane, causing a swift and excessive release of neurotransmitters, such as acetylcholine, norepinephrine, and dopamine, resulting in intense neuroexcitatory symptoms. Structural investigations utilizing electron cryo-microscopy have shown that the α-LTX monomer features a three-part structure: the wing (which includes the majority of domain II), the body (which contains part of domain II along with the beginning ankyrin repeats), and the head (created by the C-terminal ankyrin repeats) [42]. Alongside α-LTX, the venom also includes the crustacean-specific α-latrocrustatoxin (α-LCT), which has a comparable pore-forming mechanism but selectively targets crustacean neurons [37]. Additionally, low-molecular-weight peptides, LMWP and LMWP2, referred to as latrodectins, have been identified as auxiliary venom components that can enhance LTX activity, although their mechanisms of action remain incompletely understood [43]. They can also enhance the neurotoxicity of α-LTX in insect systems [8,45,46]. Phylogenetic and structural studies indicate that latrodectins originated from the ecdysozoan neuropeptide superfamily, which encompasses crustacean hyperglycemic hormones and ion transport peptides [44].

While many biochemical investigations have concentrated on *L. tredecimguttatus*, existing information indicates that the toxin repertoire is widely preserved within the *Latrodectus* genus. The detection of α-LTX, α-LIT, α-LCT, and δ-LIT in *L. tredecimguttatus* suggests a sophisticated venom profile developed to affect various prey types. Molecular information obtained from cDNA and genomic sequencing reinforces the perspective that various *Latrodectus* species produce orthologous LTXs exhibiting significant structural conservation [40,41]. Notably, several studies indicate that auxiliary elements such as LMWP and LMWP2 could influence host specificity and broaden the effects of toxins beyond their initial targets. Additionally, other venom components, including kininase, a thiol endopeptidase, and two peptides that enhance bradykinin activity, have been identified in *L. tredecimguttatus* venom [47,48]. These peptides demonstrate pharmacological properties such as enhancing bradykinin, stimulating histamine release, and inducing hypotension in vertebrates, further emphasizing the pharmacological intricacies of widow spider venom. In spite of these advancements, numerous venom components are still uncharacterized, suggesting that the complete toxicological and therapeutic potential of *Latrodectus* venom has not been thoroughly investigated.

### 2.2. Non-Venom Toxins and Toxin-like Proteins in Eggs and Spiderlings of L. tredecimguttatus

Eggs and early developmental stages of *L. tredecimguttatus* harbor a distinct repertoire of proteinaceous toxins and bioactive peptides that has been investigated alongside the better-characterized toxins of adult venom [49,50,51]. Studies of egg and spiderling extracts have identified several bioactive proteins, although their detection in developmental-stage extracts does not establish their presence in adult venom or delivery during a bite. These developmental-stage-associated components have been characterized using complementary experimental approaches, including conventional protein purification, mass spectrometry, transcriptomic profiling, and recombinant expression systems [52,53,54]. Reported egg-associated proteins include Latroeggtoxin-I through Latroeggtoxin-VI, while transcriptomic analyses have identified additional putative toxin-related sequences [52,55]. Together, these findings provide a basis for describing developmental-stage-associated protein diversity; their relevance to adult envenomation and therapeutic immunoglobulins requires separate evidence.

Available molecular evidence suggests that proteins characterized from eggs of *L. tredecimguttatus* differ from the major toxins of adult venom. Canonical α-LTXs have not been detected in the egg-derived transcriptomic and proteomic datasets discussed here; however, non-detection in these datasets does not establish their absence under all developmental conditions [52]. Biochemical studies have also described egg-associated proteins with molecular characteristics distinct from those of adult α-LTX [49,50,51,54]. The detection of toxin-related transcripts in eggs supports expression during early development, but does not exclude maternal deposition of proteins or establish that every transcript produces a functional toxin [52]. These findings distinguish egg-associated proteins from established adult venom components without demonstrating their involvement in bite-associated envenomation or their relevance as antivenom targets.

Experimental studies have documented the biological activity of egg and spiderling extracts and individual egg-associated proteins of *L. tredecimguttatus*. Comparative biochemical investigations examined venom, hemolymph, and egg extracts [56], while proteomic analysis identified substantial differences between the protein compositions of eggs and adult venom [57]. Egg and newborn spiderling extracts inhibited neuromuscular transmission in isolated mouse preparations and voltage-activated ion currents in rat dorsal root ganglion neurons [58,59]. Among individual proteins, Latroeggtoxin-II selectively inhibited tetrodotoxin-resistant sodium currents without significantly affecting tetrodotoxin-sensitive currents [60]. Latroeggtoxin-III exhibited insecticidal activity, whereas Latroeggtoxin-IV inhibited all five bacterial species tested, with the greatest activity against *Staphylococcus aureus* [50]. These findings demonstrate heterogeneous biological activities in experimental systems. However, they do not establish that the egg-associated proteins examined are present in adult venom, delivered during a spider bite, or involved in human envenomation. Their relevance as targets of therapeutic immunoglobulins therefore remains unestablished.

At the molecular level, characterized egg-associated toxins of *L. tredecimguttatus* vary considerably in molecular size and sequence. Reported components include the approximately 3.6 kDa Latroeggtoxin-IV, the 23.752 kDa Latroeggtoxin-I, and the approximately 36 kDa Latroeggtoxin-III [49,50]. N-terminal sequencing of Latroeggtoxin-I by Edman degradation revealed no significant similarity to proteins in the databases examined, highlighting the limited sequence-level characterization of some egg-associated toxins [49]. Transcriptomic and bioinformatic studies have expanded this molecular inventory: Latroeggtoxin-V was assigned to the ATPase inhibitor protein family, while transcriptomic screening identified approximately 280 unigenes with homology to known toxin families [51,52]. Such homology-based assignments identify candidate toxin-related sequences but do not establish the biological activity of every encoded protein. Pull-down assays combined with mass spectrometry identified 164 proteins associated with Latroeggtoxin-VI in PC12 cells, suggesting interactions with diverse cellular processes [54]. Further work linked its anti-inflammatory and indirect neuroprotective effects to suppression of NF-κB signaling in experimental models [55]. At the whole-extract level, SDS-PAGE analysis revealed abundant egg proteins at approximately 60 and 130 kDa, although their abundance and molecular size alone do not establish that they are toxins [57]. Together, these findings indicate considerable molecular diversity among egg-associated proteins and toxin candidates, while leaving many components insufficiently characterized at the structural and functional levels.

Taken together, studies of *L. tredecimguttatus* eggs and spiderlings reveal diverse bioactive proteins. Purification and biochemical characterization support the identity and activity of Latroeggtoxin-I [49]. Transcriptomic homology identifies candidate toxin-related sequences without establishing their functional activity [52]. Proteomic detection likewise does not demonstrate toxicity [57], and the effects of whole egg and spiderling extracts cannot be attributed to every detected protein [58,59]. Comparative proteomics characterizes differences between spiderlings and adults [61], while analysis of egg membrane proteins expands the developmental protein inventory [62]. Regulatory and neuroprotective effects reported for Latroeggtoxin-VI further emphasize the distinction between biological activity and toxicity [54,55]. Detection in eggs or spiderlings does not establish presence in adult venom, delivery during a bite, or involvement in human envenomation. These findings therefore do not justify adding developmental proteins to antivenom immunization mixtures without evidence of toxicological relevance and antibody-mediated neutralization.

## 3. Molecular Mechanisms of α-LTX Action and Receptor-Mediated Neurotransmitter Release

Early experiments demonstrated that purified α-LTX stimulates acetylcholine and norepinephrine release from mouse cerebral cortex slices [63]. Subsequent structural analysis showed that divalent cations promote α-LTX tetramer formation and that these tetramers form membrane pores [64]. Structural studies employing complementary biochemical methods, cryo-electron microscopy (cryo-EM), and high-resolution molecular reconstruction have provided increasingly detailed insight into the organization of α-LTX and the structural features underlying its interactions with presynaptic receptors [65,66,67]. At the monomeric level, α-LTX exhibits a modular architecture consisting of a structurally organized N-terminal region followed by an extended C-terminal array of ankyrin-like repeats, which are well-established motifs involved in protein–protein interactions [65,66]. High-resolution cryo-EM reconstructions and corresponding atomic models have further resolved the N-terminal portion into three structurally distinct domains connected to the extensive ankyrin-repeat region, thereby providing a structural framework for understanding receptor recognition and subsequent membrane-associated activity [66,67]. In parallel, antibody epitope-mapping and domain-fitting experiments have helped define functionally relevant regions within both the N- and C-terminal portions of the toxin [65,68]. Collectively, these structural observations indicate that α-LTX is organized into specialized molecular regions capable of coordinating receptor engagement, oligomerization, and membrane interactions rather than functioning as a structurally uniform neurotoxic protein.

This modular architecture enables α-LTX to interact with three major classes of presynaptic receptors that differ substantially in their molecular organization and dependence on extracellular Ca^2+^. Neurexins, particularly neurexin Iα, are neuronal single-pass transmembrane adhesion proteins that recognize α-LTX in a Ca^2+^-dependent manner. Early binding experiments using synaptosomal preparations identified extremely high-affinity, Ca^2+^-sensitive α-LTX-binding sites, with apparent dissociation constants (K_D) reported within the range of 0.3–5 × 10^−10^ M [69,70]. In contrast, latrophilin (ADGRL1), historically referred to as CIRL, belongs to the adhesion G-protein-coupled receptor family and undergoes autoproteolytic processing to generate an extracellular N-terminal fragment (NTF) associated with a membrane-embedded C-terminal fragment (CTF) [71,72,73]. Unlike neurexin-mediated recognition, α-LTX binding to latrophilin occurs with high affinity independently of extracellular Ca^2+^ and involves defined extracellular receptor regions, including the p85/p120-associated domains whose organization is maintained within the membrane environment [73]. RPTPσ represents a third α-LTX receptor and belongs to the receptor-type protein tyrosine phosphatase family. Although receptor-screening studies have established its capacity to interact with α-LTX, its binding properties and precise contribution to toxin-induced synaptic responses remain less extensively characterized than those of neurexin and latrophilin [67,74,75].

Importantly, these receptor interactions appear to contribute to α-LTX activity through more than simple toxin attachment to the neuronal surface. Presynaptic receptors can recruit and concentrate α-LTX at sites associated with neurotransmitter release, thereby facilitating subsequent membrane interactions, while receptor engagement can also initiate intracellular signaling events. This signaling function is particularly relevant to latrophilin, through which α-LTX can engage G-protein-dependent pathways associated with secretory responses. Consequently, the biological outcome of α-LTX exposure is determined by an interplay between receptor-mediated toxin recruitment, membrane pore formation, and intracellular signaling rather than by a single uniform mechanism. The relative contribution of these processes may vary according to receptor expression and cellular context, thereby influencing whether direct pore-mediated ionic flux or receptor-dependent signaling becomes the predominant driver of neurotransmitter release [76,77,78].

The conversion of soluble α-LTX into a membrane-inserted pore involves a divalent-cation-dependent conformational transition and oligomerization process [65,66,71]. In the absence of divalent cations, α-LTX predominantly forms dimers, whereas physiological concentrations of Ca^2+^ or Mg^2+^ promote assembly into C4-symmetric tetramers representing the active pore-forming state [65,66]. Cryo-EM analyses reveal a central axial channel and a hydrophobic membrane-facing region within these tetramers [65,68], while high-resolution structures indicate that pore formation involves coordinated rearrangement of four N-terminal helical bundles into an extended coiled-coil stalk that positions membrane-inserting helices within the lipid bilayer [66,68]. This reorganization generates a cation-permeable pore with an estimated diameter of approximately 11–13.5 Å [68]. Consistent with this model, membrane-insertion experiments in synaptosomes demonstrated protease-resistant N-terminal regions following toxin incorporation [78], and structural studies identified higher-order tetrameric assemblies with potentially distinct pore conformations [68]. Electrophysiological recordings further confirm that α-LTX forms rectifying channels permeable to monovalent and divalent cations [65,71,79], with reported single-channel conductances ranging from approximately 120–208 pS to 400 pS depending on the experimental system [68,79]. These channels are sensitive to La^3+^ and extracellular divalent cation concentrations, while α-LTX-mediated Mn^2+^ influx provides additional evidence of divalent-cation permeability [69,71]. Although receptors such as neurexin and latrophilin facilitate toxin recruitment and accelerate pore formation, the intrinsic conductance properties of the resulting channel appear to be primarily determined by the membrane-inserted α-LTX tetramer itself [71,79].

The cation-permeable pores formed by α-LTX tetramers promote substantial Ca^2+^ influx, thereby triggering neurotransmitter release through both classical and non-classical mechanisms [71,80,81,82]. In neuronal preparations, pore-mediated Ca^2+^ entry activates conventional Ca^2+^-dependent synaptic vesicle exocytosis, as demonstrated by α-LTX-induced acetylcholine release accompanied by increased intracellular Ca^2+^ in cortical synaptosomes [82], as well as glutamate and GABA release in hippocampal and neuronal models [77,81,83]. However, genetic studies indicate that α-LTX can also induce Ca^2+^-dependent exocytosis independently of key components of the canonical SNARE machinery. Specifically, substantial transmitter release persists in neurons lacking synaptobrevin/VAMP, SNAP-25, or Munc13-1, suggesting that the pronounced local Ca^2+^ elevation generated by α-LTX pores can engage alternative fusion mechanisms [80]. In addition, small neurotransmitters may undergo non-vesicular efflux directly through toxin-induced channels [80,84,85]. Consistent with these parallel mechanisms, La^3+^ suppresses the pore-mediated component by blocking α-LTX channels [69,77], whereas botulinum neurotoxins that disrupt SNARE-dependent vesicular exocytosis do not abolish non-vesicular transmitter efflux through α-LTX pores [72].

In addition to pore formation, α-LTX stimulates receptor-dependent signaling that mobilizes intracellular Ca^2+^ and promotes exocytosis through G-protein-coupled pathways [72,74,81]. This mechanism is mediated primarily by latrophilin, an adhesion GPCR that, following α-LTX binding, activates heterotrimeric G proteins and phospholipase C (PLC), resulting in PIP_2_ hydrolysis and generation of IP_3_ and diacylglycerol [72,73,74]. IP_3_ subsequently promotes Ca^2+^ release from endoplasmic reticulum stores, a process supported by studies showing sensitivity of α-LTX-induced secretion to PLC inhibition and thapsigargin [72,74]. Importantly, the pore-deficient mutant LTXN4C retains latrophilin binding and can increase presynaptic Ca^2+^, enhance spontaneous and miniature neurotransmitter release, and stimulate insulin secretion despite lacking functional pore formation, demonstrating that receptor signaling can independently promote secretion [74,77,81,84]. Unlike the pore-mediated pathway, this receptor-driven mechanism requires the canonical SNARE proteins synaptobrevin/VAMP and SNAP-25 and is partially dependent on Munc13-1 [80]. Receptor identity further influences these responses: neurexin predominantly facilitates toxin recruitment and pore-dependent Ca^2+^ entry, whereas latrophilin supports both α-LTX binding and downstream G-protein signaling [71,72,74]. RPTPσ may additionally interact with latrophilin and influence receptor-associated signaling, although its precise contribution to α-LTX-induced exocytosis remains incompletely defined [75,76].

α-LTX induces the release of diverse neurotransmitters and secretory products across neuronal and endocrine systems through combined pore- and receptor-mediated mechanisms [72,74,77,81,83,84,85]. Documented responses include acetylcholine release from cortical synaptosomes associated with intracellular Ca^2+^ elevation [82], norepinephrine secretion involving extracellular and intracellular Ca^2+^ and latrophilin/PLC signaling [72,74], dopamine release from PC12 cells accompanied by Ca^2+^ influx and PIP_2_ hydrolysis [69], and glutamate and GABA release from hippocampal preparations [77,81,83]. Beyond classical neurotransmitters, α-LTX also stimulates insulin secretion from pancreatic β-cells through contributions from both pore-dependent Ca^2+^ entry and latrophilin-mediated signaling [74,84]. This broad secretory activity reflects the toxin’s ability to engage fundamental exocytotic mechanisms: pore formation provides a direct source of Ca^2+^ that promotes vesicle fusion, whereas receptor-dependent signaling influences vesicle priming and fusion through the SNARE machinery and associated regulatory proteins [72,80]. The relative contribution of these pathways varies among cell types and may also include non-vesicular efflux of small neurotransmitters through α-LTX-induced pores [72,74,75]. Consequently, α-LTX-mediated secretion is influenced by receptor density and subtype, intracellular Ca^2+^ availability, and the composition of SNARE and regulatory proteins within individual cellular systems [77,78,80]. Collectively, these findings support a dual-mechanism model of α-LTX action in which direct pore formation and receptor-mediated signaling operate as distinct but interconnected pathways regulating neurotransmitter release. The principal experimental approaches that have established these mechanisms, together with their major findings and mechanistic implications, are summarized in Table 2.

**Figure 3:**
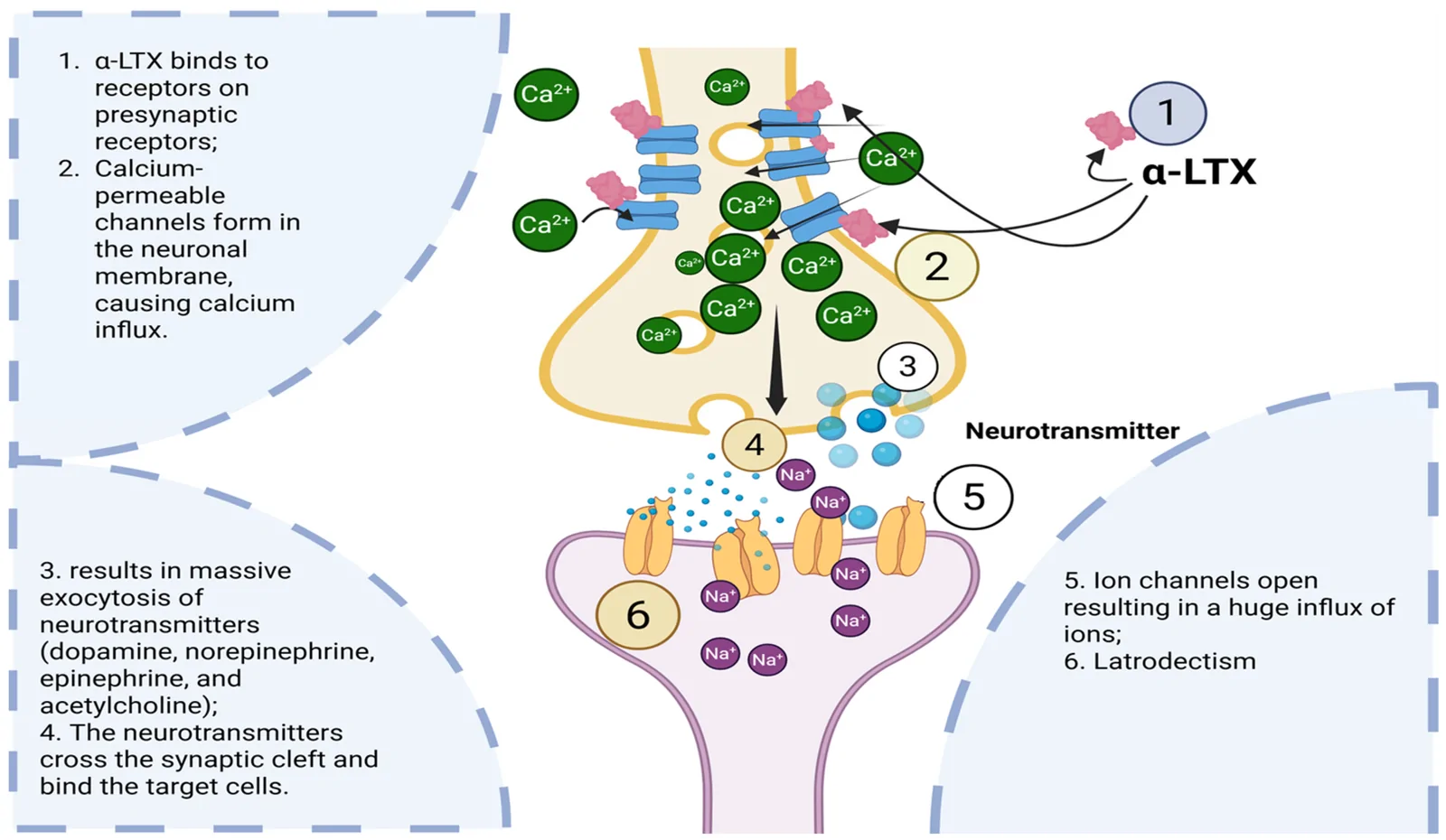
Schematic representation of α-LTX action at the presynaptic terminal. Numbered steps illustrate the sequence of toxin binding, ion flux, neurotransmitter release, postsynaptic signaling, and the resulting toxic effects (original schematic synthesized from published sources [74,77,78,80,81]).

Current evidence supports a dual-pathway model of α-LTX action in which pore formation and receptor-mediated signaling act in parallel to promote neurotransmitter release (Figure 3). In the pore-dependent pathway, α-LTX is recruited to the presynaptic membrane through receptors such as neurexin and latrophilin [69,70,73], after which Ca^2+^ or Mg^2+^ promotes its assembly into C4-symmetric tetramers [65,66]. Subsequent conformational rearrangement and membrane insertion generate cation-permeable pores [66,67,78], allowing substantial extracellular Ca^2+^ influx that drives classical vesicular exocytosis, SNARE-independent release under conditions of high local Ca^2+^, and potentially direct efflux of small neurotransmitters [71,80,82,85]. In parallel, α-LTX binding to latrophilin activates heterotrimeric G proteins and PLC, leading to PIP_2_ hydrolysis, IP_3_ production, and mobilization of Ca^2+^ from intracellular stores [72,73,74]. This receptor-mediated pathway primarily engages SNARE-dependent exocytosis involving synaptobrevin/VAMP, SNAP-25, and Munc13-1 [72,74], while RPTPσ may provide an additional modulatory contribution [75,76]. The two mechanisms can be experimentally distinguished by their pharmacological and molecular requirements, with pore-mediated responses being La^3+^-sensitive and receptor signaling being sensitive to PLC inhibition, thapsigargin, and disruption of SNARE-dependent exocytosis [69,71,72,74,77,80]. Their relative contributions vary according to cellular context, receptor expression, intracellular Ca^2+^ availability, and exocytotic machinery, while studies using the pore-deficient LTXN4C mutant further demonstrate that receptor signaling can stimulate neurotransmitter release independently of pore formation [74,77,78,80,81].

The molecular basis of α-LTX activity is well established at the level of its two principal mechanisms, although several aspects remain unresolved. Structural, biochemical, and electrophysiological studies demonstrate that Ca^2+^/Mg^2+^-dependent tetramerization and membrane insertion generate cation-permeable pores characterized by defined conductance properties and sensitivity to La^3+^ [65,66,67,68,71,78,79]. In parallel, α-LTX binding to latrophilin activates G-protein/PLC signaling and mobilizes intracellular Ca^2+^, while studies with the pore-deficient LTXN4C mutant confirm that receptor-dependent neurotransmitter release can occur independently of pore formation [72,73,74,77,81]. These pathways are further distinguished by their dependence on the exocytotic machinery, as receptor-mediated release requires canonical SNARE proteins, whereas massive pore-mediated Ca^2+^ influx can support SNARE-independent release [80]. Nevertheless, the structural determinants of α-LTX recognition by neurexin, latrophilin, and RPTPσ; the precise functional contribution of RPTPσ; and the factors governing the relative dominance of pore versus receptor signaling across cell types remain incompletely defined [67,75,76,77,78]. Moreover, the functional significance of alternative tetrameric conformations and variable pore properties [68], as well as the molecular basis of SNARE-independent transmitter release, require further investigation [80,85].

These mechanistic insights also provide a framework for developing and evaluating α-LTX-neutralizing immunoglobulins. Because α-LTX can promote neurotransmitter release through both pore formation and receptor-mediated signaling, effective antibodies should ideally interfere with molecular events common to or critical for both pathways [65,66,72,77]. Structural studies identify regions associated with oligomerization, membrane insertion, and receptor interactions as potential neutralization targets [65,67]; accordingly, antibodies that prevent tetramerization or membrane insertion could suppress pore formation, whereas antibodies that interfere with receptor engagement could reduce toxin recruitment and downstream signaling [73,77]. Pore-deficient variants such as LTXN4C may also provide useful experimental tools for investigating antibodies directed against receptor-interacting regions without retaining normal pore-forming activity [77,81]. Furthermore, established mechanistic probes, including La^3+^, PLC inhibitors, and botulinum neurotoxins, provide complementary approaches for determining whether candidate antibodies preferentially inhibit pore-dependent or receptor-mediated effect [65,72,74,77]. Thus, defining the structural and functional determinants shared between these pathways may facilitate the rational development of more potent and mechanistically comprehensive α-LTX-neutralizing immunoglobulins.

## 4. Development and Production of Specific Immunoglobulins Against *L. tredecimguttatus* Toxins

**Figure 4:**
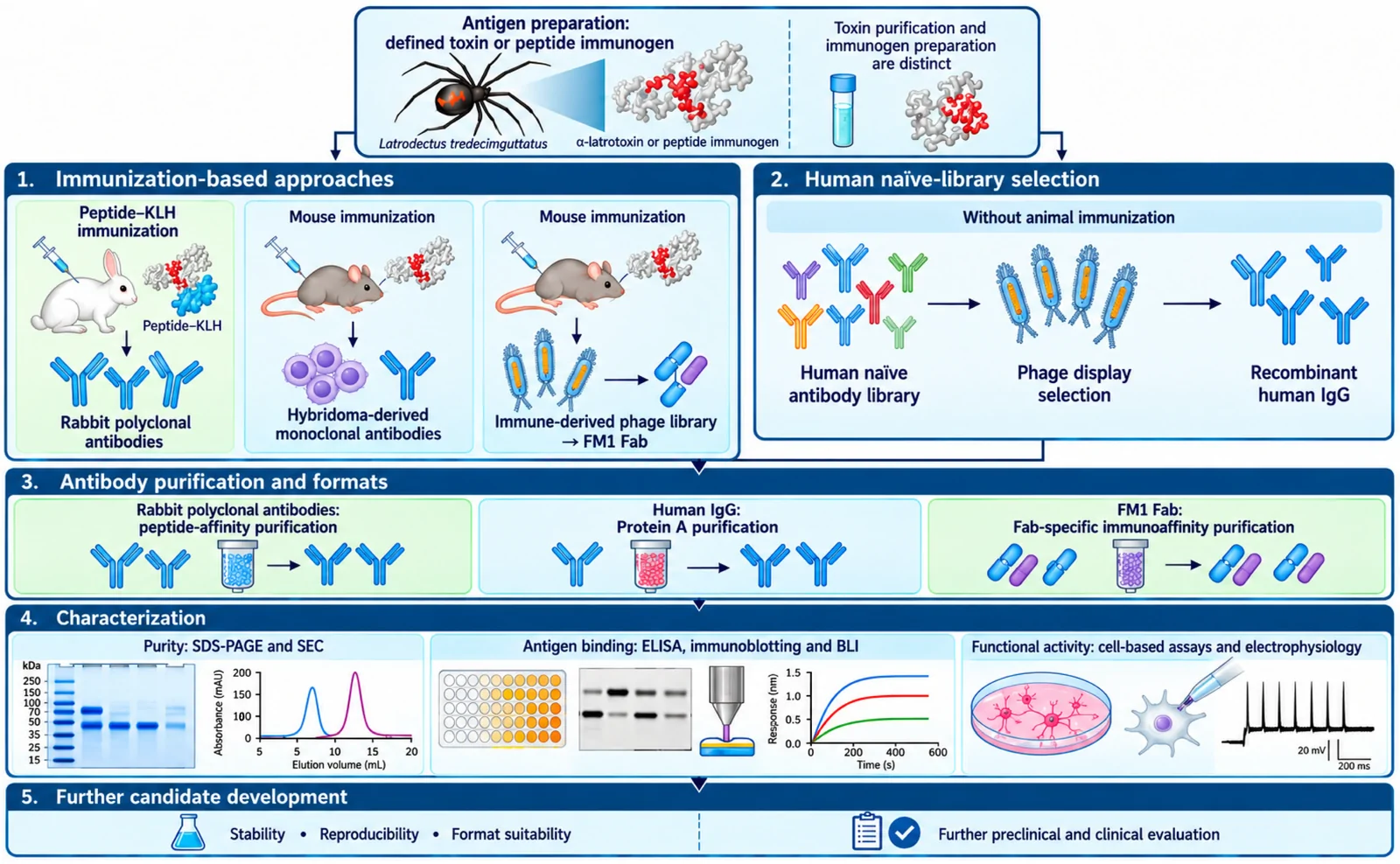
Conceptual workflow for the development of toxin-specific immunoglobulins against *L. tredecimguttatus* toxins. Immunization-based approaches include peptide-directed rabbit polyclonal antibodies [90], immune-library-derived murine FM1 Fab [95], and hybridoma-derived monoclonal antibodies [96]. Human naïve-library phage display enables antibody selection without animal immunization and subsequent production as recombinant human IgG [93,94]. The workflow distinguishes antibody purification, assessment of purity, antigen binding, and functional activity, followed by further candidate development. This original schematic illustrates research approaches rather than an established therapeutic manufacturing process. Abbreviations: KLH, keyhole limpet hemocyanin; IgG, immunoglobulin G; Fab, antigen-binding fragment; SDS-PAGE, sodium dodecyl sulfate–polyacrylamide gel electrophoresis; SEC, size-exclusion chromatography; ELISA, enzyme-linked immunosorbent assay; BLI, bio-layer interferometry.

The development of toxin-specific immunoglobulins against *L. tredecimguttatus* toxins encompasses immunization-based approaches and recombinant antibody selection. Conventional animal-derived antivenoms, including those developed against *L. hasselti*, provide a broader context for these approaches [86]. Biochemical investigations have addressed the purification of α-LTX receptors [87] and the isolation and purification of individual venom components, particularly α-LTX [88,89]. However, toxin purification should be distinguished from the demonstrated use of an antigen for immunization, as illustrated by antibodies generated against a latrodectin-derived peptide [90]. Additional context includes patent-described approaches [91] and studies involving the venomous spider *Atrax robustus*, which should be distinguished from direct evidence concerning *L. tredecimguttatus* [92]. More recently, advances in recombinant DNA technology and in vitro antibody selection have enabled the generation of fully human monoclonal antibodies through human naïve phage display libraries, thereby eliminating the requirement for animal immunization [93,94]. Overall, the production of toxin-specific immunoglobulins against *L. tredecimguttatus* involves antigen preparation, immunization or in vitro antibody selection, antibody purification and characterization, and further development of selected candidates (Figure 4).

Antigen selection is a key factor in defining the specificity of immunoglobulins developed against *L. tredecimguttatus* toxins. Whole-venom immunization provides broader antivenom-production context, including preparations developed against *L. hasselti*, rather than direct evidence of equine antiserum production against *L. tredecimguttatus* [86]. Studies of α-LTX receptor purification concern toxin–receptor interactions rather than the selection of immunogens [87]. The isolation and purification of native α-LTX, including immunoaffinity chromatography using antibody-based sorbents, provide methods for obtaining defined toxin preparations; these procedures alone do not demonstrate their use or effectiveness as immunogens [88,89]. Recombinant approaches have further expanded antigen characterization, including baculovirus-derived latrodectin. In the same study, rabbit antibodies were generated against a synthetic C-terminal latrodectin peptide conjugated to keyhole limpet hemocyanin (KLH), distinguishing recombinant protein expression from the antigen actually used for immunization [90]. Bacterially expressed N-terminal α-LTX polypeptides have also been described in a patent-based approach and should be identified as such [91]. Related peptide-based work involving *Atrax robustus* provides context from another spider species and should not be attributed to latrodectin or *L. tredecimguttatus* [92]. Thus, antigen selection should consider specificity, structural integrity, epitope preservation, and immunogenicity, while neutralizing activity requires separate functional evaluation.

The selection of an immunization host or antibody library influences the origin and characteristics of antibodies developed against *L. tredecimguttatus* toxins. Rabbits have been used to generate polyclonal antibodies against a synthetic C-terminal latrodectin peptide conjugated to KLH [90]. More recently, phage display technology has enabled the selection of fully human monoclonal antibodies from human naïve libraries without animal immunization [93,94]. This approach differs from the generation of FM1, a murine antibody selected from an immune-derived phage display library [95]. Murine models have also contributed through hybridoma technology, enabling the generation of α-LTX-specific monoclonal antibody panels [96]. Therefore, host immunization, immune-library-derived antibody selection, and human naïve-library selection represent distinct approaches whose antibody origins should be clearly distinguished.

Antibody purification should be distinguished from toxin purification and from the selection of an antibody format. Immunosorbents described for α-LTX isolation concern toxin purification rather than the purification of therapeutic immunoglobulins [88]. For selective antibody purification, peptide-affinity chromatography has been used to isolate rabbit antibodies against a C-terminal latrodectin peptide using the immobilized synthetic peptide as the affinity ligand [90]. Phage display-derived human monoclonal antibodies have been produced in mammalian expression systems and purified as recombinant IgG molecules using Protein A affinity chromatography [93,94]. Size-exclusion chromatography and SDS-PAGE were used to assess the purity of the recombinant IgG preparation [94]. The immune-library-derived murine antibody FM1 was expressed in *Escherichia coli* as a recombinant Fab fragment and purified by immunoaffinity chromatography using immobilized Fab-specific anti-mouse antibodies [95]. Thus, these studies illustrate distinct purification approaches for peptide-specific polyclonal antibodies, recombinant human IgG, and murine Fab fragments, without establishing the superiority of any particular format.

Comprehensive characterization is essential for assessing the purity, binding properties, and functional activity of *L. tredecimguttatus*-specific immunoglobulins during antibody development and production. Western blotting has been used to examine antigen recognition by peptide-specific rabbit antibodies [90]. SDS-PAGE and size-exclusion chromatography assess antibody purity and sample homogeneity. ELISA evaluates antigen binding, whereas bio-layer interferometry provides binding kinetics and K_D. ELISA-derived EC_50_ values should not be equated with K_D. Functional testing separately examines antibody effects on toxin activity using cell-based assays and electrophysiological measurements [93,94]. Western blotting, immunofluorescence, and immunocytochemical analyses provide complementary information on antigen recognition and localization under different experimental conditions [96,97]. Broader antivenom research provides additional context through reviews of alternative immunization strategies [98] and studies of plasma-derived antibody purification [99]. Patent-described antibody-fragment preparation methods represent a separate source of methodological proposals [100]. The biosynthetic oligoclonal antivenom concept was proposed for snakebite. Its applicability to *L. tredecimguttatus* remains unestablished [101]. Studies of α-LTX-specific murine monoclonal panels provide further examples of defined antibody reagents [102]. The antibody-generation approaches and formats provide a framework for comparing these candidates during further development (Table 3). Recombinant antibodies offer a defined molecular composition, but their stability, production reproducibility, and suitability in the selected format require further evaluation [93,94].

## 5. Neutralization Efficacy and Preclinical Evaluation of Specific Immunoglobulins Against *L. tredecimguttatus* Toxins

**Figure 5:**
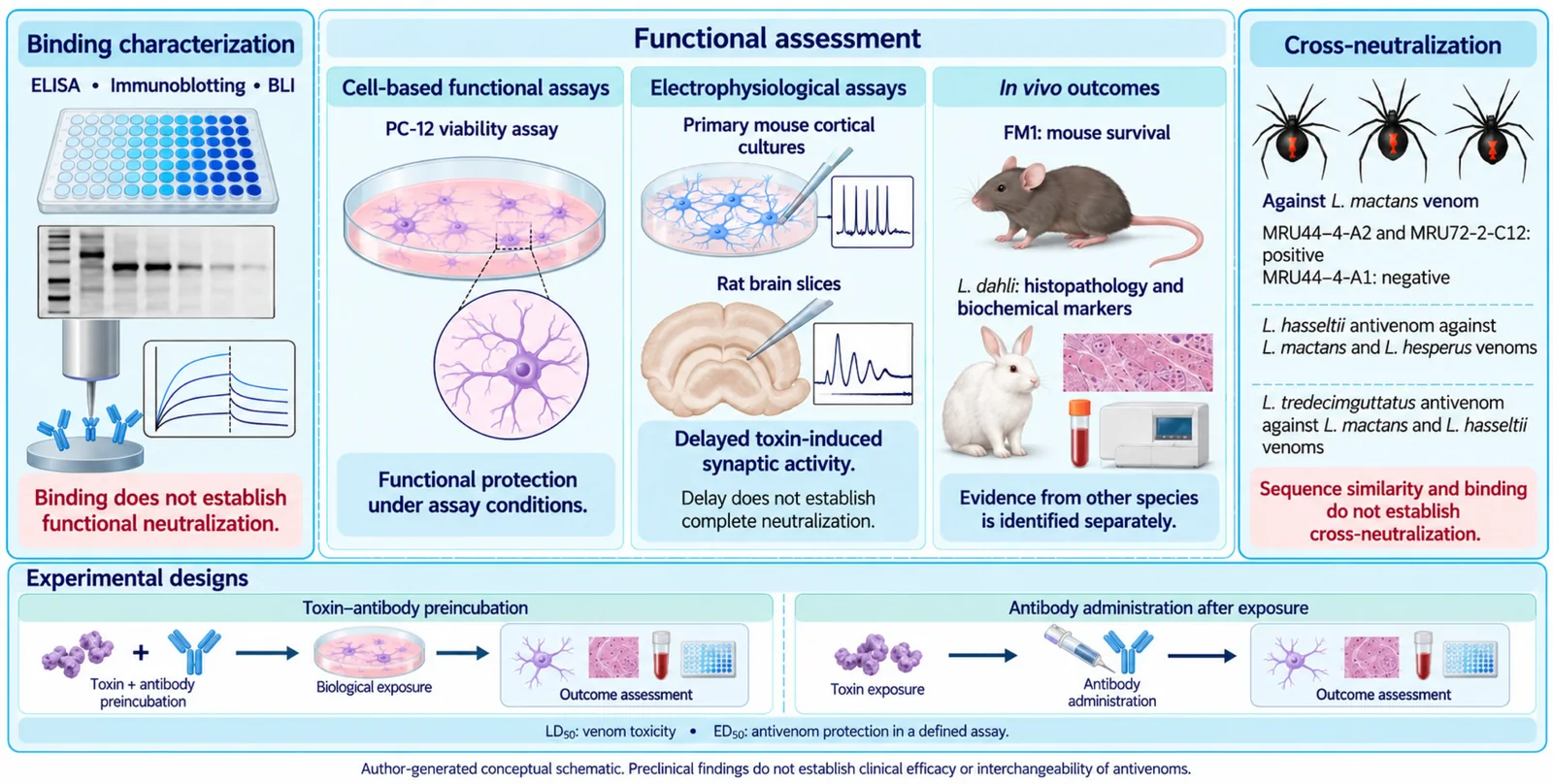
Complementary approaches to the preclinical assessment of toxin-specific antibodies against *L. tredecimguttatus* and venoms from other *Latrodectus* species. Binding characterization is distinguished from cell-based functional assays [93], electrophysiological measurements [93,94], and animal outcomes, including FM1-mediated survival in mice [95] and organ-specific findings in *L. dahli*-envenomed rabbits [108,110]. Cross-neutralization is presented as a separate experimental question [93,109,111]. Toxin–antibody preincubation and antibody administration after exposure represent distinct experimental designs. The panels do not imply a hierarchy of evidence, and delayed toxin-induced activity does not establish complete neutralization. Author-generated conceptual schematic; illustrations and traces are schematic rather than experimental data. Preclinical findings do not establish clinical efficacy or interchangeability of antivenoms. Abbreviations: ELISA, enzyme-linked immunosorbent assay; BLI, bio-layer interferometry; LD_50_, median lethal dose; ED_50_, median effective dose.

Neutralization efficacy against *L. tredecimguttatus* toxins can be evaluated using complementary experimental approaches whose relevance depends on the endpoint, experimental design, and treatment timing (Figure 5). Binding assays such as ELISA and immunoblotting establish antigen recognition but do not demonstrate functional neutralization [93]. Direct functional evidence includes cell-based assays, electrophysiological measurements in neural preparations, and mouse experiments with the murine FM1 antibody fragment [93,94,95]. Research on elapid snake venoms provides a methodological example of a toxin–receptor binding-inhibition assay correlated with mouse lethality and antivenom neutralization. However, this assay concerns α-neurotoxin–nicotinic acetylcholine receptor interactions and is not a validated assay for α-LTX [103,104]. Studies of Argentine *Latrodectus* venoms provide additional evidence of antivenom neutralization in other species [105]. Historical comparisons of black widow antisera also examined neutralizing activity, although they should be interpreted within their original experimental conditions [106]. Investigations of toxin-induced synaptic vesicle depletion characterize toxin activity rather than antibody-mediated protection [107]. Studies of *L. dahli* envenomation have assessed histopathological changes following antivenom treatment, providing evidence from another species rather than direct evidence concerning *L. tredecimguttatus* [108]. Cross-neutralization represents a separate question, examined experimentally with *L. hasselti* antivenom against *L. mactans* and *L. hesperus* venoms [109]. Biochemical markers have also been examined following antivenom treatment in *L. dahli*-envenomed rabbits, although the absence of a parallel untreated group limits attribution of the changes to treatment [110]. Cross-neutralization has additionally been investigated with *L. tredecimguttatus* antivenom against other *Latrodectus* venoms [111]. These findings do not establish clinical interchangeability. Direct evidence concerning *L. tredecimguttatus* and evidence from other species are presented separately, with attention to experimental endpoints and treatment timing (Table 4 and Table 5).

Cell-based assays provide a practical means of quantifying functional neutralization of *L. tredecimguttatus* α-LTX under controlled conditions. An alamarBlue viability assay in PC12 neurosecretory cells identified MRU44–4-A1 as a recombinant human antibody with an IC_50_ of 0.30 nM. This value describes functional protection in the assay and should be distinguished from binding measurements. The experiment used toxin–antibody preincubation before cell exposure, so the result does not establish efficacy when the antibody is administered after intoxication has begun [93]. However, cell-based models do not fully reflect the complexity of intact neural tissues or variations in receptor expression and toxin sensitivity.

Electrophysiological models allow direct measurement of antibody effects on α-LTX-induced synaptic activity in primary neuronal cultures and brain slices. In primary mouse cortical cultures, MRU44–4-A1 delayed toxin-induced increases in miniature EPSC frequency. In some recordings, the frequency did not reach the predefined threshold during the observation period [93]. In rat medial prefrontal cortex slices, whole-cell voltage-clamp recordings from pyramidal neurons showed that the recombinant human IgG TPL0020_02_G9 delayed the onset of α-LTX activity from 5.8 ± 0.8 to 19.1 ± 4.8 min and prolonged the time required to reach a four-fold increase in spontaneous EPSC frequency from 12.2 ± 3.9 to 24.6 ± 3.1 min. These experiments used a 1:10 toxin-to-antibody weight ratio with 2 h of preincubation before tissue exposure [94]. These findings demonstrate delayed toxin-induced activity rather than complete neutralization. Toxin–antibody preincubation also limits their interpretation as evidence of post-exposure treatment efficacy. Although these models capture functional synaptic responses, their interpretation remains limited by tissue viability, species-specific differences, and the absence of the pharmacokinetic and tissue-distribution processes present in intact organisms.

In vivo experiments with FM1 Fab provide direct preclinical evidence of protection against *L. tredecimguttatus* venom-gland extract under two distinct treatment conditions. In the preincubation experiment, all eight mice receiving the extract–FM1 mixture survived, whereas all eight extract-only controls died. In a separate post-exposure experiment, FM1 was administered 45 min after the extract, following the onset of clinical symptoms. All eight FM1-treated mice survived and recovered within 48 h, whereas all eight extract-only controls died within 24 h [95]. These findings demonstrate protection in both preincubation and post-exposure designs, but they do not establish a general therapeutic window or clinical efficacy. The experimental conditions and survival outcomes are presented separately (Table 4).

Evidence from *L. dahli* envenomation should be distinguished from direct findings concerning *L. tredecimguttatus*. In New Zealand white rabbits, antivenom administered 24 h after venom injection reduced severe cardiac histopathological lesions. However, mild myocytolysis and hemorrhage persisted in one treated rabbit, indicating incomplete tissue protection [108]. A separate biochemical study reported decreases of 47.58% in AST and 65.88% in ALP at 48 h after antivenom administration, relative to post-venom, pre-antivenom values. Changes in renal biochemical markers were also reported. The absence of a parallel untreated envenomed group limits attribution of these changes to antivenom rather than spontaneous recovery [110]. These organ-specific findings do not establish efficacy against *L. tredecimguttatus* venom or clinical benefit in humans (Table 5).

Cross-neutralization among *Latrodectus* species varies considerably and requires functional testing rather than inference from toxin sequence similarity or antibody binding. Among fourteen recombinant human antibodies against *L. tredecimguttatus,* α-LTX, MRU44–4-A2 and MRU72–2-C12 neutralized *L. mactans* whole venom in the cell-based assay. MRU44–4-A1 did not show cross-neutralization under the tested conditions [93]. *L. hasselti* antivenom also protected mice against *L. mactans* and *L. hesperus* venoms in experiments using venom–antivenom preincubation. These results establish cross-neutralization in that experimental design but do not establish post-exposure efficacy [109]. Early studies demonstrated that *L. tredecimguttatus* antivenom could protect against *L. mactans* and *L. hasselti* venoms in mice following post-exposure administration [111]. The reported venom LD_50_ values characterize toxicity rather than antivenom neutralizing potency [109]. Neither sequence similarity nor binding alone establishes functional cross-neutralization, and animal protection does not establish clinical interchangeability. Polyvalent formulations therefore remain a proposal for investigation rather than a demonstrated means of achieving broader protection (Table 5).

Overall, preclinical studies demonstrate progress in evaluating antibody-mediated protection against *L. tredecimguttatus* toxins through cell-based assays, electrophysiological models, and in vivo experiments. Recombinant human antibodies offer a defined molecular composition and represent preclinical candidates for further development. Their superiority over conventional antivenoms, clinical efficacy, and safety remain unestablished [93,94]. Nevertheless, translation of these candidates remains challenging, particularly because neutralizing activity can vary between experimental models and cross-neutralization is antibody-dependent [93]. Protection under preincubation conditions does not establish equivalent efficacy after envenomation. Further evaluation should address post-exposure efficacy, the therapeutic window, pharmacokinetics, and the optimal antibody format. It should also determine whether a single antibody provides sufficient protection and whether selected candidates cover the toxic effects of whole venom. Direct comparison among studies remains difficult because of differences in cellular and animal models, assay endpoints, venom preparations, and experimental protocols [93,94,95]. Future preclinical studies should evaluate models that better reflect clinically relevant venom effects and validate functional assays for the specific venom–antibody combinations under investigation [112]. Standardized protocols and transparent reporting of venom challenge doses, administration routes, assay endpoints, and neutralization potency units are also needed to support reliable comparisons across studies [113].

## 6. Clinical Applications and Therapeutic Use

Antivenom provides a specific therapeutic approach to latrodectism, although its clinical benefits and risks require critical assessment [28]. Clinical experience with *L. mactans* antivenom primarily concerns North American black widow envenomation [114]. Randomized studies of redback spider antivenom concern *L. hasselti* and must be interpreted within their respective populations and treatment comparisons [115,116]. Neither evidence base independently establishes efficacy against *L. tredecimguttatus* or clinical interchangeability between antivenom products. Direct clinical evidence for Mediterranean latrodectism remains predominantly observational. In a retrospective Croatian cohort, 21 antivenom-treated patients had median durations of severe and moderate pain after administration of 1.2 and 2.4 h, respectively, and a median hospitalization of 4 days. Shorter pain duration and hospitalization were reported compared with untreated patients, but the nonrandomized design remains susceptible to treatment-selection bias and confounding [117]. Additional clinical experience has been reported in Turkey [118], Greece [119], and Spain [120]. These publications inform clinical presentation and management but do not establish standardized antivenom indications or administration protocols.

Experimental cross-neutralization represents a separate category of evidence. Redback spider antivenom protected mice against *L. mactans* and *L. hesperus* venoms after venom–antivenom preincubation [109]. This demonstrates neutralization under the tested conditions, without establishing post-exposure efficacy in humans or efficacy against *L. tredecimguttatus*. A report of F(ab′)_2_ antivenom treatment for *L. geometricus* concerns another species [121]. In contrast, an Italian case described improvement following treatment of *L. tredecimguttatus* envenomation with North American *L. mactans* antivenom [122]. This provides direct clinical experience with a heterologous preparation, but a single uncontrolled observation cannot establish reproducible efficacy, clinical interchangeability, or a standardized administration protocol.

The RAVE study was a randomized, double-blind, double-dummy trial comparing intravenous (IV) and intramuscular (IM) redback spider antivenom in 126 patients with latrodectism. The primary outcome was clinically significant pain improvement 2 h after the last treatment. Improvement occurred in 40/64 IV recipients (62%) and 33/62 IM recipients (53%), with a reported difference of 9 percentage points favoring IV administration (95% Bayesian credible interval: −8 to +26 percentage points). The posterior probability of any IV advantage was 85%, but the probability of exceeding the prespecified clinically important difference of 20 percentage points was only 10%. The interval included no difference, and the Bayesian analysis did not support a clinically important IV advantage; these probabilities are not frequentist *p* values. Both groups received antivenom, preventing assessment of efficacy against supportive care alone. The 24 h pain findings were secondary outcomes [116].

RAVE-II was a multicenter, randomized, double-blind, placebo-controlled trial involving 224 patients aged ≥8 years with redback spider envenomation. Following standardized analgesia, patients received two vials of IV antivenom or placebo. The primary pain outcome, clinically significant pain reduction at 2 h, occurred in 38/112 antivenom recipients (34%) and 26/112 placebo recipients (23%): a difference of 10.7 percentage points favoring antivenom (95% confidence interval: −1.1 to 22.6; *p* = 0.10). The second primary outcome, resolution of systemic effects at 2 h among patients with systemic envenomation, occurred in 9/35 (26%) and 9/41 (22%), respectively: a difference of 3.8 percentage points (95% confidence interval: −15 to 23; *p* = 0.79). Neither primary outcome nor the secondary outcomes differed significantly between groups. The trial did not demonstrate additional benefit over standardized analgesia, although a small benefit could not be excluded [115].

Administration evidence must be distinguished from clinical efficacy. A pharmacokinetic substudy of RAVE measured serum antivenom concentrations in 20 patients. Antivenom was detected in all 10 IV recipients but remained undetectable in the samples collected from the 10 IM recipients [123]. These findings describe systemic exposure to the tested preparation and do not independently establish clinical superiority or define administration protocols for *L. tredecimguttatus*. As a substudy of RAVE, this publication should not be counted as an independent clinical trial.

Evidence from pediatric populations also requires species-specific interpretation. An Australian retrospective study reported a good clinical effect in 57/61 children (93%) treated with redback spider antivenom [124]. This uncontrolled assessment concerns *L. hasselti* and cannot establish pediatric efficacy against *L. tredecimguttatus*. Differences in species, preparations, populations, and study designs limit comparisons with the Mediterranean clinical reports.

Safety findings require attribution to the administered preparation and study population. In RAVE-II, acute hypersensitivity reactions occurred in 4/112 antivenom recipients (3.6%) and were described as mild, skin-only reactions [115]. Adverse events following North American black widow antivenom have been examined separately in a different clinical setting [125]. Neither dataset establishes adverse-reaction rates for heterologous treatment of *L. tredecimguttatus*. Likewise, antibody fragmentation or recombinant human antibody design alone does not demonstrate superior clinical safety.

**Figure 6:**
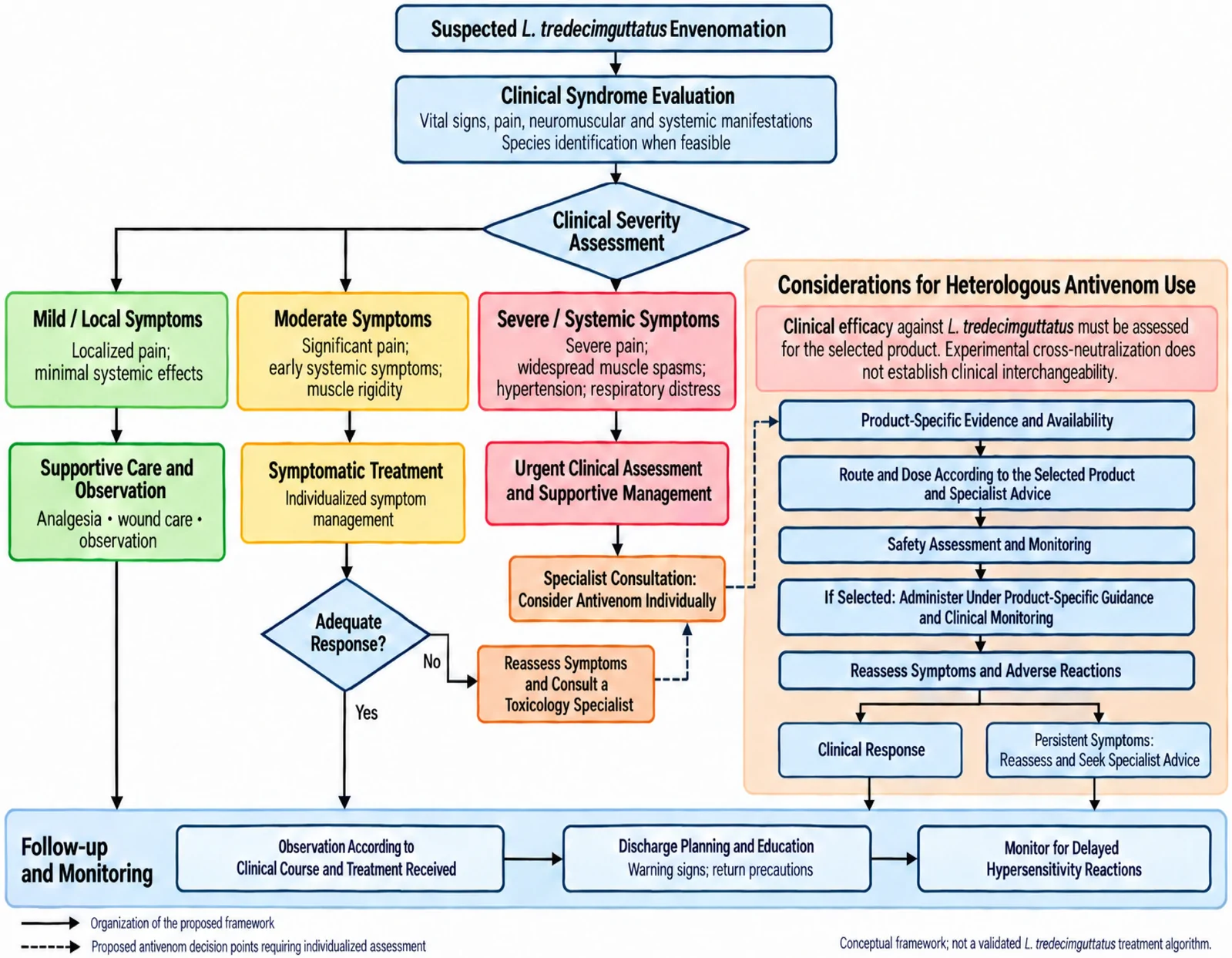
Conceptual framework for clinical assessment and management of *Latrodectus tredecimguttatus* envenomation. Original schematic synthesized from clinical reports concerning *L. tredecimguttatus* [117,118,119,120,122] and potentially informative studies of redback spider antivenom [115,116]. The framework has not been validated as a species-specific treatment algorithm. Solid arrows indicate the organization of the framework; dashed arrows indicate antivenom decision points requiring individualized assessment. Antivenom selection and administration require product-specific evaluation. Experimental cross-neutralization does not establish clinical interchangeability.

Overall, direct clinical observations, experimental cross-neutralization, and clinical findings from other *Latrodectus* species provide distinct categories of evidence. Phylogenetic relatedness, toxin similarity, and serological cross-reactivity do not establish functional neutralization or clinical interchangeability. Clinical assessment, supportive management, reassessment of persistent symptoms, and individualized consideration of a defined antivenom product are organized within a conceptual framework (Figure 6). This synthesis has not been validated as a species-specific treatment algorithm. Future studies should evaluate defined products in confirmed *L. tredecimguttatus* envenomation using standardized efficacy and safety outcomes.

## 7. Current Challenges, Limitations, and Future Perspectives

A central challenge in antibody development for *L. tredecimguttatus* is defining the antigen coverage required to neutralize clinically relevant venom effects. Recombinant antibodies against α-LTX have demonstrated functional neutralization in preclinical assays [93]. However, whether targeting α-LTX alone provides sufficient protection against different whole-venom preparations and exposure conditions remains unresolved. Comparative proteomics has identified differences between spiderlings and adults, but these observations do not establish the contribution of developmental proteins to human envenomation [61]. Their inclusion in immunization mixtures would require evidence of delivery during a bite, clinically relevant toxicity, and inadequate neutralization by existing preparations. Antigen selection should therefore prioritize experimentally demonstrated contributions to envenomation and antibody-mediated protection.

Antibody selection requires functional testing and mechanistic characterization. Cross-neutralization of *L. mactans* venom by antibodies selected against *L. tredecimguttatus* α-LTX was antibody-dependent, demonstrating that neutralizing coverage cannot be inferred from toxin similarity alone [93]. Earlier monoclonal antibody experiments identified a functional domain involved in toxin-induced secretion that was distinct from the toxin’s receptor-binding site [96]. Future studies should combine neutralizing epitope mapping and structural analysis of antibody–toxin complexes with assays examining receptor engagement, oligomerization, and pore formation. These approaches should establish which functional steps individual antibodies inhibit. Whether complementary antibodies provide broader or more effective protection than a single candidate requires direct comparison against defined venom preparations.

Product definition and manufacturing consistency are additional development priorities. Defined recombinant antibody mixtures have been proposed in snake antivenom development, but their applicability to *L. tredecimguttatus* requires separate experimental evaluation [101]. The identification of a human α-LTX-binding antibody [94] and subsequent demonstration of functional neutralization by selected candidates [93] provide a basis for further development. Optimization should establish specifications for antibody identity, purity, aggregation, formulation stability, and functional potency. Changes in affinity or antibody format should be evaluated for their effects on neutralization and retention of activity during production and storage. Improved safety, scalability, or batch consistency should not be assumed from recombinant design alone.

Clinical translation requires evidence beyond protection under toxin–antibody preincubation conditions [93]. Future studies should evaluate post-exposure treatment against defined whole-venom preparations, establish the therapeutic window, and assess pharmacokinetics and pharmacodynamics to inform antibody format and dosing. Standardized assay conditions and transparent reporting of challenge doses, administration routes, endpoints, and potency units are also needed to support comparisons across studies, consistent with methodological recommendations developed for snake antivenom testing [113]. Direct clinical experience with heterologous antivenom in *L. tredecimguttatus* envenomation remains observational [122]. Prospective, preferably controlled studies should therefore evaluate defined preparations in patients with documented species identification using prespecified efficacy and safety endpoints. Clinical efficacy and safety of recombinant candidates remain unestablished.

## 8. Conclusions

*L. tredecimguttatus* envenomation represents a clinically important neurotoxic condition in which α-LTX is a principal molecular target for antibody development. Advances in toxin characterization and understanding of α-LTX receptor interactions, oligomerization, membrane insertion, and pore formation have provided a mechanistic foundation for the rational development of neutralizing antibodies. Conventional animal-derived polyclonal antivenoms provide a specific treatment option for latrodectism, although their clinical benefits depend on the species, preparation, and treatment setting. Their use is constrained by the risk of adverse reactions and limited regional availability, while direct clinical evidence for *L. tredecimguttatus* remains predominantly observational. Recombinant human monoclonal antibodies represent an important step toward more defined antibody preparations, with experimental studies demonstrating functional α-LTX neutralization by selected candidates. Nevertheless, antibody binding, functional neutralization, and clinical efficacy represent distinct outcomes. Preclinical activity should not yet be interpreted as established clinical efficacy, particularly because cross-neutralization can be incomplete, post-exposure effectiveness remains insufficiently characterized, and direct clinical evidence for *L. tredecimguttatus* is limited. Findings from other *Latrodectus* species and experimental cross-neutralization do not establish clinical interchangeability of antivenoms. Thus, further development of recombinant candidates will require standardized functional assays, clinically relevant post-exposure models, pharmacokinetic evaluation, and controlled clinical validation. Overall, integrating mechanistic knowledge of α-LTX with recombinant antibody engineering and rational selection of complementary neutralizing specificities provides a basis for further investigation of immunoglobulin-based therapies for *L. tredecimguttatus* envenomation; improved safety, reproducibility, and broader protection remain objectives requiring demonstration.

## Figures and Tables

**Table 1:** Comprehensive overview of major venom components isolated from *L. tredecimguttatus*.

Component	MW (kDa)	Primary Target	Experimentally Supported Characteristics	Refs.
α-LTX	130	Vertebrates	Stimulates neurotransmitter release; forms membrane pores following tetramerization. Structural analysis describes wing, body, and head regions.	[35]
α-LIT	120	Insects	Purified toxin increases miniature excitatory postsynaptic potential frequency in insect neuromuscular preparations. Forms ion-conducting channels in artificial lipid bilayers.	[36]
β-LIT	140	Insects	Isolated insect-active latrotoxin. A specific receptor and detailed mechanism should not be assigned from studies of other LITs.	[36]
γ-LIT	120	Insects	Isolated insect-active latrotoxin; reported activity depends on the experimental species and preparation.	[36]
δ-LIT	Approximately 100–110, mature toxin	Insects	Cloning identified an ankyrin-repeat-containing precursor. C-terminal truncation was required for functional recombinant expression; functional experiments demonstrated insect-specific activity and channel formation.	[36]
α-LCT	120	Crustaceans	Crustacean-active latrotoxin; cryo-EM analysis resolved its monomeric architecture.	[37]
ε-LIT	110	Insects, C. elegans	Insect-active latrotoxin; toxicity to *Caenorhabditis elegans* is reported in the cited review. Its mechanism should not be inferred from α-LIT or δ-LIT.	[36,38]
LMWP	Approximately 8	Auxiliary enhancer	Co-purifies with α-LTX and exhibits structural similarity to crustacean hyperglycemic hormones. Recombinant studies examined its influence on toxin activity.	[43,44]
LMWP2	Approximately 9.5	Auxiliary enhancer	Characterized low-molecular-weight venom component. Evolutionary analyses support membership of latrodectins in the CHH/ITP neuropeptide superfamily.	[43]

**Table 2:** Experimental evidence for the molecular mechanisms of α-LTX action.

Experimental Approach	Key Findings	Mechanistic Insight	Pore vs. Receptor Pathway	Refs.
Cryo-EM structural determination	α-LTX forms C4-symmetric tetramers with central axial channel in presence of Ca^2+^/Mg^2+^; two distinct tetramer conformations observed with pore diameters ~11–13.5 Å	Tetrameric assembly creates cation-permeable channel; divalent cations stabilize pore-forming oligomer	Pore formation	[68]
Biochemical membrane insertion assay	N-terminal fragment of α-LTX becomes protease-resistant upon incubation with synaptosomes at physiological temperature	α-LTX inserts into membrane with N-terminal region exposed to cytoplasm	Pore formation	[78]
Patch-clamp electrophysiology	α-LTX induces rectifying cationic currents permeable to mono- and divalent cations; La^3+^ blocks currents; single-channel conductances of ~120–400 pS depending on preparation	Direct demonstration of functional ion channel formation; pore provides Ca^2+^ entry route	Pore formation	[71,79]
Mn^2+^ quenching of fura-2 fluorescence	α-LTX mediates Mn^2+^ entry into cells, detected by fura-2 quenching	Confirms divalent cation permeability through α-LTX pores	Pore formation	[69,71]
Receptor binding studies in synaptosomes	High-affinity α-LTX binding sites with Kd ~0.3–5 × 10^−10^ M; Ca^2+^-dependent (neurexin) and Ca^2+^-independent (latrophilin) populations	Identifies distinct receptor classes that recruit toxin to presynaptic sites	Both pathways (receptor recruitment)	[69,70,73]
Latrophilin/CIRL functional characterization	Latrophilin is adhesion-class GPCR; α-LTX binding activates G proteins and PLC, leading to IP_3_ generation and Ca^2+^ release from stores	Receptor-mediated signaling mobilizes intracellular Ca^2+^ independently of pore formation	Receptor signaling	[72,73,74]
PLC inhibitor and thapsigargin experiments	PLC inhibitors and thapsigargin block α-LTX-evoked norepinephrine release from nerve terminals	Demonstrates requirement for PLC signaling and intracellular Ca^2+^ stores in receptor-mediated pathway	Receptor signaling	[72,74]
Mutant toxin LTXN4C studies	LTXN4C retains receptor binding but lacks pore activity; stimulates Ca^2+^ mobilization from stores and enhances spontaneous release in La^3+^-insensitive manner	Dissociates receptor signaling from pore formation; proves receptor pathway can trigger release independently	Receptor signaling	[74,77,81]
SNARE protein knockout/knockdown	Pore-mediated (neurexin-anchored) α-LTX-evoked exocytosis proceeds in neurons lacking synaptobrevin/VAMP, SNAP-25, or Munc13-1; receptor-mediated pathway requires these SNAREs	Defines SNARE-independent (pore/Ca^2+^-flood) versus SNARE-dependent (receptor-signaling) exocytosis pathways	Pore (SNARE-independent) vs. Receptor (SNARE-dependent)	[80]
Botulinum neurotoxin experiments	Botulinum neurotoxins block vesicular (SNARE-dependent) release but not non-vesicular neurotransmitter efflux through α-LTX pores	Distinguishes vesicular exocytosis from direct efflux through large pores	Both pathways (differential sensitivity)	[69,72,77]
La^3+^ blockade studies	La^3+^ blocks α-LTX-induced currents and pore-mediated release but not receptor-signaling readouts (e.g., PIP_2_ hydrolysis)	Pharmacologically separates pore-dependent from receptor-dependent mechanisms	Pore (La^3+^-sensitive) vs. Receptor (La^3+^-insensitive)	[69,71,77]
Neurotransmitter release assays (acetylcholine, norepinephrine, glutamate, GABA, dopamine, insulin)	α-LTX triggers release of diverse neurotransmitters and hormones across neuronal and endocrine preparations with varying dependencies on extracellular Ca^2+^, intracellular stores, and SNAREs	Demonstrates broad functional impact on exocytosis; cell-type differences reflect variable contributions of pore vs. receptor pathways	Both pathways (context-dependent)	[69,72,74,77,81,82,83,84]
Electrophysiology in hippocampal neurons with LTXN4C	LTXN4C increases spontaneous EPSC frequency without affecting evoked EPSCs	Receptor signaling modulates vesicle release probability independently of pore-mediated Ca^2+^ influx	Receptor signaling	[77,81]

**Table 3:** Production strategies and characterization of specific immunoglobulins against *Latrodectus* toxins.

Approach/Antibody	Antigen or Target	Antibody Source and Purification	Binding/Characterization Evidence	Functional Neutralization Evidence	Ref.
Peptide-directed polyclonal antibodies	C-terminal peptide of latrodectin conjugated to KLH	Rabbit antiserum; peptide-affinity chromatography on Sepharose	ELISA and immunoblotting; recognition of latrodectin required reducing conditions in immunoblots	Neutralization was not demonstrated; recognition of the native functional protein cannot be inferred from immunoblotting alone	[90]
Human naïve-library phage display: MRU44–4-A1 and other antibodies	α-LTX	Human antibody libraries; recombinant expression in Expi293F cells; Protein A purification	ELISA, immunoblotting, and BLI; lead antibody evaluated in IgG, scFv-Fc, and Fab formats	Cell-based and electrophysiological neutralization; two antibodies also cross-neutralized *L. mactans* venom	[93]
Human naïve-library phage display: TPL0020_02_G9	Native α-LTXs	Human scFv library; conversion to IgG1 and expression in HEK293 cells; Protein A chromatography	ELISA against toxin and whole venom; purity assessed by SDS-PAGE and SEC	Ex vivo inhibition of toxin-induced changes in synaptic activity in rat brain slices	[94]
Immune-library phage display: FM1 Fab	α-LTX	Antibody library from immunized BALB/c mice; Fab expression in *E. coli*; anti-mouse Fab immunoaffinity chromatography	ELISA and nanomolar-range toxin binding; SDS-PAGE of purified Fab	Inhibition of toxin-induced Ca^2+^ uptake; symptom remission and prevention of venom-extract lethality in experimentally envenomed mice	[95]
Monoclonal antibodies, including 4C4.1	α-LTX	Monoclonal antibody panel; production and purification details are not specified	ELISA; recognition of cell-bound toxin under specified experimental conditions	4C4.1 inhibited toxin- and venom-induced dopamine release, toxin-induced Ca^2+^ accumulation, and channel formation in artificial lipid bilayers	[96]
Monoclonal antibodies used for tissue localization	α-LTX in venom glands	Previously generated monoclonal antibodies; production details are not	Indirect immunofluorescence and immunogold electron microscopy localized toxin within venom-gland cells	Not assessed in this study	[97]
Monoclonal antibodies, including A4, A6, A15, and A24	α-LTX	Monoclonal antibody panel; production and purification details are not	Antibody effects were assessed without inhibition of toxin binding to rat synaptosomes	Antibody-dependent inhibition or modification of secretogenic and channel-forming effects in synaptosomes and lipid bilayers	[102]

**Table 4:** Preclinical functional effects and neutralization of *L. tredecimguttatus* α-LTX and venom by monoclonal antibodies.

Target Toxin/Preparation	Antibody Tested	Experimental Model	Study Design/Timing	Toxin Challenge	Antibody Dose/Ratio	Functional Endpoint and Result	Main Limitation	Ref.
Purified α-LTX	Human recombinant IgG MRU44–4-A1	PC12 cells	Toxin–antibody preincubation for 1 h at RT before cell exposure	4.2 nM α-LTX	Antibody titration: 210–approximately 0.067 nM; initial antibody:toxin molar ratio, 50:1	Cell viability measured by alamarBlue; neutralization IC_50_: 0.30 nM	Preincubation-based cellular assay; post-exposure efficacy not established	[93]
Purified α-LTX	Human recombinant IgG MRU44–4-A1	Primary mouse cortical neuronal culture	Preincubated toxin–antibody mixture applied by perfusion	1 nM α-LTX	10 nM antibody; antibody:toxin molar ratio, 10:1	Onset of increased mEPSC frequency delayed up to 18 min after wash-in; onset threshold not reached during observation in 3 of 9 recordings	Delayed onset does not establish sustained or complete neutralization; limited recording duration	[93]
Purified α-LTX	Human recombinant IgG TPL0020_02_G9	Rat medial prefrontal cortex brain slices	Toxin–antibody preincubation for 2 h at RT	Final α-LTX concentration, 1 nM	Toxin:antibody ratio, 1:10 *w*/*w*	Mean onset of increased spontaneous EPSC frequency delayed from 5.8 ± 0.8 to 19.1 ± 4.8 min	Delayed activity in an ex vivo preparation; complete neutralization not established	[94]
Unfractionated venom gland extract	Murine monoclonal Fab FM1	Mice in vivo	Extract–antibody preincubation for 5 min before IP injection	35 μg extract/mouse; described as LD_100_ under the experimental conditions	20 μg/mouse in Methods and Table 1 legend; 25 μg in Results ^1^	Survival at 24 h: 8/8 FM1-treated mice versus 0/8 mice receiving extract without FM1	Optimized preincubation; internally inconsistent antibody dose	[95]
Unfractionated venom gland extract	Murine monoclonal Fab FM1	Mice in vivo	Antibody administered IP 45 min after extract injection, following symptom onset	35 μg extract/mouse	25 μg/mouse	Survival: 8/8 FM1-treated mice versus 0/8 mice receiving extract without FM1; recovery reported within 48 h	Small experimental groups; a single post-exposure treatment delay	[95]

Numerical values are reported directly in the cited sources; none were independently calculated or extracted from figure curves. All toxin preparations in this table originate from *L. tredecimguttatus*. ^1^ For FM1 preincubation, the original study [95] reports inconsistent amounts of 20 μg and 25 μg in different parts of the publication. The ELISA binding EC_50_ of 0.09 nM for MRU44–4-A1 is distinct from its functional neutralization IC_50_ and is therefore excluded from the functional results column. No neutralization of *L. mactans* venom was demonstrated for MRU44–4-A1 [93]. These preclinical findings do not establish clinical efficacy or clinical interchangeability of antivenoms. Abbreviations: α-LTX, α-latrotoxin; IgG, immunoglobulin G; Fab, antigen-binding fragment; RT, room temperature; IP, intraperitoneal; EPSC, excitatory postsynaptic current; mEPSC, miniature excitatory postsynaptic current; IC_50_, half-maximal inhibitory concentration; EC_50_, half-maximal effective concentration; LD_100_, dose producing complete lethality under the stated experimental conditions; *w*/*w*, weight-to-weight ratio.

**Table 5:** Preclinical antivenom studies involving other *Latrodectus* species and cross-species neutralization.

Target Species/Preparation	Antivenom Tested	Experimental Model	Study Design/Timing	Venom Challenge and Toxicity Characterization	Antivenom Dose	Functional Endpoint and Result	Cross-Neutralization Evidence	Main Limitation	Ref.
*L. dahli* crude venom	Specific *Latrodectus* antivenom supplied by the Razi Institute	New Zealand white rabbits in vivo	Antivenom administered IV 24 h after venom injection	0.5 mg/kg SC	2.5 mL; stated neutralizing capacity, 500 LD_50_/mL ^1^	Histopathology: severe cardiac lesions were reduced; mild myocytolysis and hemorrhage persisted in one treated rabbit. No numerical neutralization estimate reported	Not assessed	Incomplete tissue protection; no direct evidence for *L. tredecimguttatus*	[108]
*L. mactans* and *L. hesperus* venoms	*L. hasselti* antivenom	Mice in vivo; randomized, double-blind experiment	Venom–antivenom preincubation for 1 h at 25 °C before IP injection	Actual challenge dose unavailable in the supplied abstract. Venom LD_50_: 0.26 mg/kg for *L. mactans* and 0.64 mg/kg for *L. hesperus* ^2^	Unavailable in the supplied abstract	Survival at 24 h: all venom–antivenom mice survived, whereas all venom–protein-control mice died in both experiments; *p* < 0.0001	Demonstrated against both *L. mactans* and *L. hesperus* venoms in this mouse model	Optimized preincubation; post-exposure efficacy not established	[109]
*L. dahli* crude venom	Specific *L. dahli* antivenom	New Zealand white rabbits in vivo	Antivenom administered IV 24 h after venom injection	0.5 mg/kg SC	2.5 mL; stated neutralizing capacity, 500 LD_50_/mL ^1^	At 48 h after antivenom administration, AST decreased by 47.58% and ALP by 65.88% relative to post-venom, pre-antivenom values; both *p* < 0.01	Not assessed	Serial measurements without a parallel untreated envenomed group; changes cannot be attributed exclusively to antivenom	[110]
*L. mactans* and *L. hasselti* venom-containing cephalothoracic extracts	Sheep antiserum raised against *L. tredecimguttatus* venom; produced in Yugoslavia	White mice in vivo	Antiserum administered IP 30 min after extract injection; survival assessed at 24 h	Challenge expressed as fractions of venom gland contents; potency experiment used two certain lethal doses of *L. mactans*	For the *L. mactans* potency experiment: 0.1 mL of a 1:1 dilution. Comparative experiments against both species used 0.5 mL	Complete protection against two certain lethal doses of *L. mactans* at the stated diluted-antiserum dose; protection also reported against *L. hasselti*	Demonstrated against *L. mactans* and *L. hasselti*	Historical extract preparation and gland-based dose units; limited comparability with modern standardized assays	[111]

Numerical values are reported directly in the cited sources; none were independently calculated or extracted from figure curves. Studies involving other *Latrodectus* species do not establish neutralization of *L. tredecimguttatus* venom. ^1^ The stated 500 LD_50_/mL describes antivenom potency reported by the source, rather than an efficacy estimate derived from the rabbit experiment [108,110]. ^2^ Venom LD_50_ values characterize venom toxicity; they are neither antivenom neutralization potency estimates nor the actual challenge doses [109]. These preclinical findings do not establish clinical efficacy or clinical interchangeability of antivenoms. Abbreviations: IP, intraperitoneal; SC, subcutaneous; IV, intravenous; LD_50_, median lethal dose; AST, aspartate aminotransferase; ALP, alkaline phosphatase.

## Data Availability

No new data were created or analyzed in this study. Data sharing is not applicable to this article.

## Abbreviations

The following abbreviations are used in this manuscript:

LCT	α-Latrocrustatoxin
α-LTX	α-Latrotoxin
ADGRL1	Adhesion G protein-coupled receptor L1
ALP	Alkaline phosphatase
AST	Aspartate aminotransferase
BLI	Bio-layer interferometry
cDNA	Complementary DNA
CIRL	Calcium-independent receptor for α-latrotoxin
cryo-EM	Cryo-electron microscopy
CTF	C-terminal fragment
DNA	Deoxyribonucleic acid
EC_50_	Half-maximal effective concentration
ED_50_	Median effective dose
ELISA	Enzyme-linked immunosorbent assay
EM	Electron microscopy
EPSC	Excitatory postsynaptic current
Fab	Antigen-binding fragment
F(ab′)_2_	Bivalent antigen-binding fragment
GABA	γ-Aminobutyric acid
GPCR	G protein-coupled receptor
IC_50_	Half-maximal inhibitory concentration
IgG	Immunoglobulin G
IM	Intramuscular
IP	Intraperitoneal
IP_3_	Inositol 1,4,5-trisphosphate
IV	Intravenous
K_D	Equilibrium dissociation constant
KLH	Keyhole limpet hemocyanin
LD_50_	Median lethal dose
LD_100_	Dose producing complete lethality under the stated experimental conditions
LIT	Latroinsectotoxin
LMWP	Low-molecular-weight peptide
LMWP2	Low-molecular-weight peptide 2
LTX	Latrotoxin
mEPSC	Miniature excitatory postsynaptic current
MW	Molecular weight
NF-κB	Nuclear factor κB
NTF	N-terminal fragment
pI	Isoelectric point
PIP_2_	Phosphatidylinositol 4,5-bisphosphate
PLC	Phospholipase C
RAVE	Redback Antivenom Evaluation
RAVE-II	Second Redback Antivenom Evaluation study
RPTPσ	Receptor-type protein tyrosine phosphatase σ
RT	Room temperature
SC	Subcutaneous
SDS-PAGE	Sodium dodecyl sulfate–polyacrylamide gel electrophoresis
SEC	Size-exclusion chromatography
SNAP-25	Synaptosomal-associated protein of 25 kDa
SNARE	Soluble N-ethylmaleimide-sensitive factor attachment protein receptor
VAMP	Vesicle-associated membrane protein
*w*/*w*	Weight-to-weight ratio
